# Help-seeking among women with disabilities who experience domestic violence in Uganda: evidence from UDHS 2006, 2011, and 2016

**DOI:** 10.1080/16549716.2026.2640684

**Published:** 2026-03-09

**Authors:** Kirabo Suubi, Fredrik Norström, Suzanne Namusoke Kiwanuka, Fredinah Namatovu

**Affiliations:** a Department of Epidemiology and Global Health, Umeå University, Umeå, Sweden; b Department of Research, International Center for Research on Women (ICRW), Kampala, Uganda; c Department of Health Policy, Planning and Management, Makerere University College of Health Sciences School of Public Health, Kampala, Uganda; d Centre for Demographic and Ageing Research (CEDAR), Umeå University, Umeå, Sweden

**Keywords:** Disability inclusion, gender-based violence, economic participation, age differences, sub-saharan Africa

## Abstract

**Background:**

Women with disabilities face heightened vulnerability to domestic violence and often encounter multiple barriers to seeking help. However, empirical evidence on help-seeking behaviour among this group in low-resource settings remains limited.

**Objective:**

The study assessed factors associated with help-seeking among women with disabilities following exposure to domestic violence in Uganda using nationally representative data.

**Methods:**

We analysed data from 2006, 2011, and 2016 Uganda Demographic and Health Surveys. Disability status was determined using Washington Group Short Set of Questions on functional difficulties. The sample comprised women with disabilities aged 15–49 who reported experiencing domestic violence. Descriptive statistics and logistic regression models estimated associations between socio-demographic characteristics and help-seeking, accounting for sampling weights, clustering, and stratification.

**Results:**

Help-seeking among women with disabilities remained low over time. In 2016, only about two in five women (43%) who experienced domestic violence reported seeking help. Employment and age were strongly associated with help-seeking. Employed women with disabilities were six times more likely to seek help than their unemployed peers (AOR = 6.68; 95% CI: 1.53–29.23). Younger women were less likely to seek help than older women. No significant associations were observed for education, wealth, or place of residence.

**Conclusions:**

Employment and older age emerged as important enablers of help-seeking among women with disabilities who are experiencing domestic violence. Strengthening and implementing age-appropriate and inclusive labour policies that promote economic participation and awareness of support services for women with disabilities may enhance their access to domestic violence services in Uganda and similar low-resource settings.

## Paper Context

- **Main findings:** Women with disabilities in Uganda experience high levels of domestic violence but face persistent barriers to accessing support services, despite progressive national legal frameworks. Using nationally representative data, this study identifies employment and older age as a key factor enabling help-seeking among women with disabilities.
- **Added knowledge:** Evidence about help-seeking among women with disabilities in low-resource settings is limited. This study adds population-level insights by showing how demographic factors shape help-seeking over time.
- **Global health impact for policy and action:** The findings highlight the need for strengthened economic opportunities for women with disabilities and age-appropriate disability-inclusive domestic violence services. These insights can guide more equitable policy and programme responses in Uganda and similar settings.

## Background

Women with disabilities (WWDs) are disproportionately affected by domestic violence (DV), facing compounded risks based on discrimination, stigma, misconceptions about disability, gender, poverty, reliance on the perpetrators, and social isolation [1–5]. DV is a persistent worldwide public health problem that is associated with significant adverse consequences [3–6]. In Uganda, the DV Act of 2010 defines DV as acts or omissions causing physical, sexual, emotional, psychological, or economic harm within domestic relationships [7]. This definition aligns with the United Nations Declaration on the Elimination of Violence against Women (1993) which defines DV as ‘any act of gender-based violence (GBV) that results in, or is likely to result in, physical, sexual, or psychological harm or suffering to women, including threats of such acts, coercion, or arbitrary deprivation of liberty, whether occurring in public or in private life’ and recognises it as a serious violation of human rights [8]. DV is considered a highly gendered social issue that disproportionately affects women [6,9]. One in three women worldwide experience physical and/or sexual intimate partner violence or non-partner sexual violence during their lifetime [10,11], with higher prevalence in Africa, where nearly half of all countries report rates of physical violence against women at over 40% [12]. In Uganda, 48% of women aged 15–49 reported experiencing either physical or sexual violence in 2022 [13]. DV is associated with severe health and social consequences, including physical and mental health problems, such as injuries, depression, post-traumatic stress disorder, and poor sexual and reproductive health outcomes, often continuing long after the abusive relationship ends [3,4,14–17].

### Women with disabilities and domestic violence

Globally, 19% of women have a disability [18]. WWDs experience up to 10 times more violence and for longer periods than those without disabilities [3,5,19]. In Uganda, 23% of women report some functional difficulty [20], and half of all WWDs have experienced physical or sexual violence [21]. Research suggests that disability is both a risk marker and a consequence of DV in women. Women reporting DV are significantly more likely to report a disability or chronic condition that impacts upon their ability to carry out daily living activities [5,22]. The WHO defines disability based on the bio-psychosocial model, in which disability is viewed as a result of the interaction between health conditions and contextual factors, including environmental and personal factors [18].

### Help-seeking after experiencing DV

Help-seeking after experiencing DV is critical for victims’ recovery and safety [23–28]. In general, women survivors of violence seek help from informal/non-institutional sources, such as family and friends, with formal help-seeking [17,29,30] from sources, such as healthcare, the police, and social services far less likely than informal help-seeking [23–25]. Cross-national studies in low- and middle-income countries (LMICs) have revealed help-seeking rates between 35% and 40% among women experiencing physical and/or sexual violence, with estimates ranging from 22.5% in Southeast Asia to 72.2% in Latin America [24]. In sub-Saharan Africa (SSA), patterns of inadequate help-seeking persist despite the high rates of DV in the region. A recent study by Dickson et al. of 24 SSA countries shows that about 61% of women do not seek help after experiencing intimate partner violence (IPV), with the highest rate in Mali at 80% [26]. Studies in Tanzania found that although 42% of women reported experiencing IPV, only half of them sought any form of help, while in Nigeria, 65% of IPV survivors did not seek assistance [27,31].

Evidence shows that, for WWDs, seeking help when experiencing violence can be a more challenging and complex process than for other women [24,25,29,32]. Existing research highlights that effective responses to violence against WWDs must account for multiple dimensions of access, including approachability, acceptability, availability, appropriateness, and affordability, as outlined in the conceptual framework developed by Levesque et al. [29,33]. Robinson et al. further demonstrate that misalignment between these accessibility needs and existing violence-related services may limit help-seeking among survivors with disabilities [34]. Furthermore, factors such as social acceptance and the normalisation of violence within communities, as well as the negative perceptions and stigma associated with abuse, often deter women from disclosing their experiences due to fear of social repercussions, and fear of jeopardising their family’s honour [24,25,28,32,35,36].

### Legal and regulatory framework in Uganda

Uganda has established legal and policy frameworks to address DV, including the 1995 Constitution of Uganda, the Prevention of Trafficking in Persons Act 2009, the DV Act 2010 [7] and its 2011 regulations, the prohibition of Female Genital Mutilation Act 2010 and its regulations, and the 2019 National Policy on the Elimination of GBV in Uganda [37], among others. Similarly, Uganda has a legal framework that protects persons with disabilities (PWDs). The Constitution (1995) provides commitments to equality for PWDs. Other policies include the Employment Act 2006, the Education Act 2008, the Persons with Disabilities Act 2020 [38], the revised National Policy for Persons with Disabilities (2023), and the National Action Plan on Disability 2023/2024–2028/2029. These policies aim to reduce vulnerabilities and improve access to support services. However, their implementation remains inconsistent due to inadequate funding, poor service delivery and monitoring, and lack of awareness among the population, among other factors [39,40]. Responses to DV involving WWDs span multiple sectors, including health services, the police and justice system, community structures, and civil society organisations [7,41]. Key interventions include facility-based survivor care, reporting through Family and Child Protection Units, legal redress under the Domestic Violence Act, and referral pathways coordinated at the community level [41].

Despite the high prevalence of DV and the compounded risks associated with it, there is very little population-based research about what drives or hinders help-seeking after violence, especially in LMICs [24,25], and particularly among WWDs, who are among the most vulnerable. This evidence gap has real consequences; without a clear understanding of the factors shaping help-seeking, policies, and services remain poorly designed, insufficiently resourced, and often inaccessible. As a result, WWDs continue to face violence with only limited pathways to safety, justice, or recovery. The current study addresses this critical gap by focusing specifically on WWDs, a group that remains significantly underserved in DV research and policy [42].

This study aims to assess the factors associated with help-seeking among WWDs following exposure to DV. The findings will inform the development of targeted interventions and policies that are responsive to the specific needs of WWDs, contributing to more inclusive, equitable, and effective strategies for DV prevention and response.

## Methods

### Study design, data, and sample

**Figure 1.**
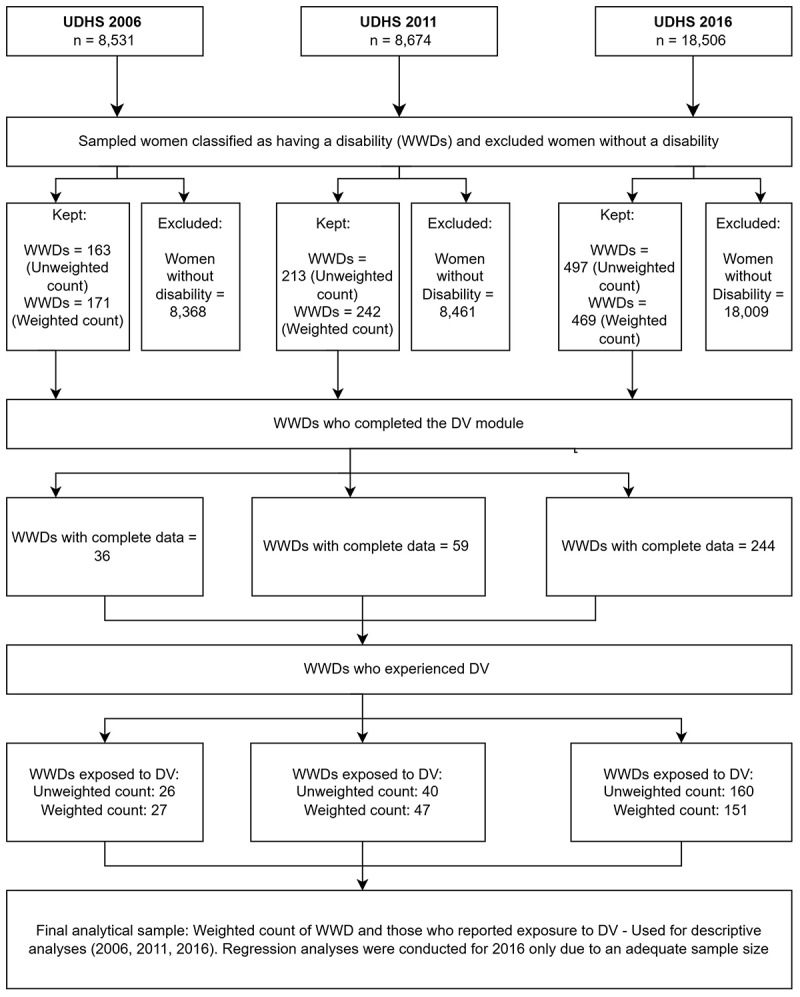
Flow chart of analytical sample (UDHS 2006–2016).

Cross-sectional data from the Uganda Demographic and Health Surveys (UDHS) in 2006, 2011, and 2016 was used [41,43,44]. The UDHS provides up-to-date estimates of key demographic, socioeconomic, and health indicators in Uganda. These specific survey years were selected because they provide the most recent publicly accessible datasets containing data on disability. The UDHS uses a stratified, two-stage sampling design to produce nationally representative estimates. The UDHS data is nationally representative for women aged 15–49 years. The surveys adhered to standardised DHS protocols, including ethical procedures for administering the DV Module, with interviews conducted only when privacy was ensured and informed consent obtained. Detailed information about the survey design is available in the UDHS final survey reports [41,43,44]. Permission to use the datasets was formally requested and granted by the DHS Programme [45] prior to analysis. This study was conducted in accordance with the ethical principles of the Declaration of Helsinki. All data was fully de-identified before release, and no personally identifiable information was available to the authors. As this study worked exclusively with anonymised secondary data, no additional ethical approval was required. The datasets were downloaded and exported to Stata version 18.5. Household member datasets were then linked with the individual women’s dataset, which includes a DV module [46]. A total of 8531 women were interviewed in 2006, 8674 in 2011, and 18,506 in 2016. More details about the study population are provided in Figure 1.

### Disability measure

We focused on WWDs, and disability status was the main exposure variable. UDHS surveys included the DHS Programme’s disability module [47], a series of questions based on the Washington Group Short Set of Questions on Disability (WGSS). These questions address the six functional domains of vision, hearing, mobility, communication, self-care, and cognition [48]. Respondents provided information as to whether they had no difficulty, some difficulty, a lot of difficulty, or cannot do at all within any of the six domains [41,43,44]. In this study, classification for disability status followed the Washington Group’s recommended ‘Disability 3’ categorisation analytical approach. Individuals were classified as having a disability if they reported ‘a lot of difficulty’ or ‘cannot do at all’ in at least one of the six functional domains; otherwise, they were defined as having no disability [22]. A supplementary file, Appendix 1, is attached to illustrate the specific questions asked. The final analytical sample comprised women who both reported exposure to DV and met the criteria for disability. See the flow chart of the analytical sample.

#### Help-seeking behaviour measure

Our primary outcome for the current study was whether a WWD who had experienced DV had ever sought help. The common sources of help were their own family, their husband’s/intimate partner’s family, friends, and chief/other national government administrative officers. The question used to screen for those who had sought help after DV was ‘thinking about what you yourself have experienced among the different things we have been talking about (DV experiences), have you ever tried to seek help?’ The participants who responded ‘yes’ were recoded as ‘1’, signifying those who sought help after DV. Participants who responded ‘no’ were recoded as ‘0’ and categorised as those who did not seek help after violence.

### Covariates

Sociodemographic variables were selected based on prior literature demonstrating their relevance to help-seeking behaviour among survivors of violence [24,49]. The variable age was categorised as 15–24, 25–34, and 35+. Education was grouped as ‘received any formal education’ (including primary, secondary, or more) and ‘no formal education’. Marital status was categorised as ‘single’ or ‘in a relationship’. Employment status (yes or no) and place of residence (urban or rural) were also recorded. Household wealth index was grouped into high, middle, or low categories based on survey-defined criteria [41,43,44].

### Statistical analysis

Descriptive statistics were used to summarise sample characteristics across survey years and to assess factors associated with help-seeking behaviour among WWDs. Odds ratios (OR) with 95% confidence intervals (CI) were calculated with logistic regression on the 2016 data to assess sociodemographic variables influencing help-seeking behaviours among WWDs. The numbers of WWDs in 2006 and 2011 were so small that they did not garner results in the regression models.

All analyses took the complex survey design of the UDHS into account by applying sample weights and adjusting for clustering and stratification using Stata version 18.5. A completecase analysis was used, with cases missing data on disability status, DV module variables, or model covariates being excluded from the analyses. The svy command was used for all weighted analyses [24]. Variables such as marital status with high multicollinearity (variance inflation factor [VIF] > 10) were excluded from the final models. A *p*-value below 0.05 was considered statistically significant.

## Results

### Socio-economic and demographic characteristics of women with disabilities in Uganda (2006, 2011, and 2016)

Table 1 presents the socio-economic and demographic characteristics of the sample of WWDs based on data from the UDHS 2006, 2011, and 2016. Over the years, mobility and cognition-related disabilities were the most common. The majority of WWDs were aged 35 years and above. More than 70% had attained some level of education, and this proportion increased by approximately 8% over the decade. Most WWDs were in a relationship and resided in rural areas. About three-quarters engaged in some form of employment in each survey year. Many of the WWDs fell within the middle wealth quintile, and this proportion increased by 18% between 2006 and 2016.

**Table 1.** Description of the sample size of WWDs in UDHS, 2006–2016.

Variables	2006 (*n* = 171)		2011 (*n* = 242)		2016 (*n* = 469)	
	Number	Weight %	Number	Weight %	Number	Weight %
**Disability type**						
Seeing	42	0.49	94	1.09	17	0.10
Hearing	17	0.20	20	0.24	2	0.01
Mobility	61	0.72	86	1.00	210	1.14
Communication	9	0.11	9	0.11	20	0.11
Self-care	10	0.12	10	0.12	25	0.14
Cognition	51	0.61	60	0.69	255	1.38
**Age groups**						
35 years and above	57	33.5	128	52.9	234	49.9
25–34	54	31.6	55	22.7	122	26.1
15–24	60	35.0	59	24.4	113	23.9
**Education**						
No formal education	45	26.6	54	22.5	85	18.2
Received any formal education	126	73.4	188	77.5	384	81.8
**Marital status**						
Single	87	50.8	103	42.6	177	37.7
In a relationship	84	49.2	139	57.4	292	62.3
**Place of residence**						
Urban	27	15.9	30	12.4	100	21.3
Rural	144	84.1	212	87.6	369	78.7
**Employment**						
No	43	24.9	72	29.9	115	24.4
Yes	128	75.1	170	70.1	354	75.6
**Wealth**						
High	48	28.1	37	15.3	70	15.1
Middle	91	53.1	155	63.8	335	71.4
Poor	32	18.8	52	20.9	64	13.6

### Factors associated with help-seeking after domestic violence among women with disabilities (2006, 2011, 2016)

Table 2 presents the distribution of WWDs who sought or did not seek help after experiencing DV, disaggregated by socio-demographic characteristics for the years 2006, 2011, and 2016. Among WWDs who responded to the DV module, 68% in 2006, 70% in 2011, and 66% in 2016 reported having experienced at least one form of DV. Among those who reported experiencing violence, the proportion who reported seeking help was 56% (15) in 2006, 54% (25) in 2011, and 43% (65) in 2016.

**Table 2.** Factors associated with help-seeking among women with disabilities who have experienced domestic violence.^a^

Years	2006 (*n* = 26)				2011 (*n* = 40)				2016 (*n* = 160)			
Variable	No help sought		Sought help		No help sought		Sought help		No help sought		Sought help	
	n	%	n	%	n	%	n	%	n	%	n	%
**Employment**												
No	1	26	2	74	5	38.6	7	61.4	16	88.6	4	11.4
Yes	11	45.2	12	54.8	12	49.4	16	50.6	78	51.8	62	48.2
**Education**												
No formal education	4	41	4	59	2	8.2	5	91.8	17	71.9	8	28.0
Received any formal education	8	44.7	10	55.3	15	53.2	18	46.8	77	53.3	58	46.7
**Wealth**												
High	2	14.9	4	85.1	6	70.7	5	29.3	9	39.5	5	60.5
Middle	7	45.9	10	54.1	9	38.7	14	61.3	66	62.4	42	37.6
Poor	3	100	0	0	2	32.2	4	67.8	19	47.4	19	52.6
**Age groups**												
35 years and above	5	44.4	5	55.6	7	37.8	15	62.2	43	47.3	42	52.7
25–34	5	27.3	9	72.7	8	63.2	4	36.8	36	71.7	17	28.3
15–24	2	100	0	0	2	51.2	4	48.8	15	72.1	7	27.9
**Place of residence**												
Urban	2	13.4	4	86.6	5	72.5	5	27.5	13	50.2	10	49.8
Rural	10	53.1	10	46.9	12	38.7	18	61.3	81	58.7	56	41.3
**Marital status**												
Single	3	33.1	7	66.9	5	49.6	9	50.4	22	49	20	51
In a relationship	9	56.9	7	43.1	12	43.5	14	56.5	72	60.7	46	39.3

^a^Used unweighted counts and weighted percentages.

Seeking help was significantly lower among the unemployed women than their employed counterparts by 2016. Nearly half of employed WWDs sought help (48%), compared with about 1 in 10 unemployed women (11%). Among those with some education, the proportion seeking help declined slightly, by about 8.6% over the decade, while declines were more pronounced among WWDs with no education (31%). A notable improvement in help-seeking after DV was observed among poor WWDs, rising from 0% in 2006 to 27.9% in 2016. Younger women (15–24 years) consistently reported the lowest rates of help-seeking compared to those aged 35 and above. The rural–urban gap of WWDs seeking help narrowed over time, with proportional differences reducing to 8.5% by 2016.

Employed WWDs were statistically significantly more likely to seek help (adjusted OR 6.68; 95% CI: 1.53–29.23), than the unemployed (Table 3). The association was not statistically significant for education, wealth, or place of residence. In the comparisons between age groups, younger WWDs were less likely to seek help than those aged 35 and above, although this was statistically significant only for women aged 25–34 (adjusted OR 0.27; 95% CI: 0.11–0.67).

**Table 3.** Factors associated with help-seeking after experiencing domestic violence among women with disabilities.

Variable	2016	
	Crude model	Adjusted model
**Employment**		
No	1	1
Yes	7.24 (1.78–29.34)	6.68 (1.53–29.23)
**Education**		
No	1	1
Yes	2.25 (0.64–7.91)	3.86 (0.89–16.61)
**Wealth**		
Highest	1	1
Middle	0.39 (0.10–1.53)	0.65 (0.12–3.56)
Poor	0.72 (0.61–3.28)	1.72 (0.20–14.77)
**Age groups**		
35 years and above	1	1
25–34	0.36 (0.13–0.91)	0.27 (0.11–0.67)
15–24	0.35 (0.09–1.21)	0.38 (0.10–1.38)
**Place of residence**		
Urban	1	1
Rural	0.71 (0.25–2.03)	0.65 (0.16–2.75)

## Discussion

This study assessed the factors associated with help-seeking behaviours among WWDs in Uganda who had experienced DV, using nationally representative DHS data from 2006, 2011, and 2016. The findings show that, although the prevalence of DV remained relatively stable across the three survey periods (68% in 2006, 70% in 2011, and 66% in 2016), help-seeking among WWDs declined steadily over the same timeframe (from 56% in 2006 to 43% in 2016). This divergence could suggest that the factors influencing help-seeking may have weakened over time, even as exposure to DV persisted at consistently high levels. These results align with prior evidence from sub-Saharan Africa showing that WWDs are disproportionately exposed to violence, yet often underreport or avoid seeking assistance [24,26]. Another study found that more than one in four WWDs who experienced violence globally either did not or would not seek any form of support, largely due to compounded structural and interpersonal barriers [32].

A key finding of our study is that, in Uganda, unemployed WWDs who have experienced DV are less likely to seek help than employed WWDs who have experienced DV. Employed WWDs had over six times the odds of seeking help compared to their unemployed peers. The results are consistent with evidence that women’s economic participation and empowerment enhance their agency, bargaining power, and autonomy in decisions related to seeking protection or justice [22,50,51]. Employment may also increase access to social networks, exposure to information, and resources that facilitate help-seeking and safer exit strategies from perpetrators [24,25,52].

Beyond employment, age emerged as an important factor in help-seeking. Our findings show that younger WWDs were less likely to seek help than those aged 35 years and above. This finding aligns with evidence from other studies in the region and broader SSA contexts, where older women were more likely to seek help after experiences of violence than younger women [26,31,53]. These age differentials suggest that help-seeking among WWDs is shaped not only by economic resources but also by life-course factors, which may reflect differences in autonomy, social networks, perceived risk, or familiarity with support systems [26].

WWDs often face social isolation, stigma, and low self-esteem [5,19] and economic participation may therefore serve as a critical entry point for strengthening help-seeking pathways among this population by reducing dependency and social isolation, and by increasing the confidence to seek help. However, recent empirical work emphasises that employment alone is not a universal remedy, and that economic autonomy may interact with household power dynamics, social norms, and local service environments in complex ways [25,52]. For WWDs, structural barriers, such as inaccessible facilities, discriminatory attitudes among service providers, and communication barriers can blunt the protective potential of employment unless services and legal mechanisms are explicitly disability-inclusive [54–57]. Studies on violence among WWDs have noted a higher prevalence of abuse and substantial obstacles to service use, even when victims have some economic resources, highlighting the need for multi-pronged, coordinated interventions that address economic, social, and institutional constraints simultaneously [29,32,55,57,58].

### Strengths and limitations

A major strength of this study is its focus on the population of WWDs, who have previously received little attention in research yet are at heightened risk of being excluded from health-seeking due to the intersecting marginalisation related to their functional limitations, gender, and other social factors. Although the small sample size constrains the generalisability of the findings, this study addresses a longstanding gap in both research and policy discourse in Uganda and other LMICs [6,16]. The findings provide insights into the situation of WWDs, which is currently missing [42] in Uganda and many low-resource settings. Moreover, this relatively small sample size is considered sufficient given the rare occurrence of the event under study [59–61]. However, we call upon other scholars with access to larger samples to investigate this association in order to better inform policy and practice. The use of repeated cross-sectional DHS data enabled an assessment of changes over time using nationally representative samples. One of the limitations of the study is that DV experiences are likely to be underreported due to stigma, fear, and social desirability bias, particularly among WWDs, which may lead to conservative estimates of prevalence and association. Trend interpretation should be made cautiously, given the small subgroup sizes. In addition, the use of secondary data restricted the study’s control over variable construction and measurement. The cross-sectional design precludes causal inferences about observed associations; therefore, all causal interpretations should be made cautiously.

## Conclusion

Based on the study’s findings, employment and age appear to be important facilitators of help-seeking for WWDs when they experience DV, although broader social, economic, and institutional contexts shape their influence. These findings underscore the need for age-responsive and disability-inclusive labour policies and strategies, especially targeted at WWDs living in low-resource settings such as Uganda. Future research should use multiple methods, including a qualitative participatory approach, to explore the quality, type, and accessibility of services, as well as the intersectional barriers that limit WWDs’ ability to seek and receive support. Further work is also needed to explore the best mechanisms through which economic participation operates, including informal employment, control over income, workplace safety, and social support networks, in order to better inform integrated and inclusive violence-response strategies.

## Supplementary Material

DHS8_Module_Disability_EN_29Jun2017_DHSQM_de.docx

## Data Availability

This study used secondary data from the Uganda Demographic and Health Surveys (2006, 2011, and 2016). These datasets are publicly available through the DHS Programme upon formal request at https://dhsprogram.com/data. The authors are not permitted to share the raw datasets but can provide analysis code upon reasonable request.
