# Supplementary material for: Help-seeking among women with disabilities who experience domestic violence in Uganda: evidence from UDHS 2006, 2011, and 2016
Source: Glob Health Action. 2026 Mar 9;19(1):2640684. doi: 10.1080/16549716.2026.2640684 (PMC12973794; doi:10.1080/16549716.2026.2640684)
Supplement: DHS8_Module_Disability_EN_29Jun2017_DHSQM_de.docx [file ZGHA_A_2640684_SM7935.docx]

FORMATTING DATE: 24 May 2016

ENGLISH LANGUAGE: 31 Jan 2017

DEMOGRAPHIC AND HEALTH SURVEYS DISABILITY MODULE

MODEL HOUSEHOLD QUESTIONNAIRE

[NAME OF COUNTRY]

[NAME OF ORGANIZATION]

| **IDENTIFICATION (1)** | | | | | | |
| --- | --- | --- | --- | --- | --- | --- |
| PLACE NAME  NAME OF HOUSEHOLD HEAD  CLUSTER NUMBER . . . . . . . . . . . . . . . . . . . . . . . . . . . . . . . . . . . . . . . . . . . . . . . . . . . . . . . . . . . . . . . . . . . . . .  HOUSEHOLD NUMBER . . . . . . . . . . . . . . . . . . . . . . . . . . . . . . . . . . . . . . . . . . . . . . . . . . . . . . . . . . . . . . . . . . .   \|  \|  \|  \|  \| \| --- \| --- \| --- \| --- \| \|  \|  \|  \|  \| \|  \| \| \|  \|   HOUSEHOLD SELECTED FOR MAN'S SURVEY? (1=YES, 2=NO) . . . . . . . . . . . . . . . . . . . . . . . . . . . . . . . . . . . . . . . . . . . . | | | | | | |
| **INTERVIEWER VISITS** | | | | | | |
|  | 1 | 2 | 3 | FINAL VISIT | | |
| DATE  INTERVIEWER'S NAME  RESULT* |  |  |  | DAY  MONTH  YEAR  INT. NO.  RESULT* | | |
| NEXT VISIT: DATE  TIME |  |  |  | TOTAL NUMBER OF VISITS | | |
| *RESULT CODES:   1. COMPLETED 2. NO HOUSEHOLD MEMBER AT HOME OR NO COMPETENT RESPONDENT   AT HOME AT TIME OF VISIT   1. ENTIRE HOUSEHOLD ABSENT FOR EXTENDED PERIOD OF TIME 2. POSTPONED 3. REFUSED 4. DWELLING VACANT OR ADDRESS NOT A DWELLING 5. DWELLING DESTROYED 6. DWELLING NOT FOUND 7. OTHER   (SPECIFY) | | | | \|  \|  \| \| --- \| --- \|   TOTAL PERSONS IN HOUSEHOLD   \|  \|  \| \| --- \| --- \|   TOTAL ELIGIBLE  WOMEN   \|  \|  \| \| --- \| --- \|   TOTAL ELIGIBLE  MEN  LINE NO. OF   \|  \|  \| \| --- \| --- \|   RESPONDENT  TO HOUSEHOLD  QUESTIONNAIRE | | |
| \| **0** \| **1** \| LANGUAGE OF INTERVIEW** \|  \|  \| NATIVE LANGUAGE  OF RESPONDENT** \|  \|  \| \| --- \| --- \| --- \| --- \| --- \| --- \| --- \| --- \|   LANGUAGE OFTRANSLATOR USED  QUESTIONNAIRE**(YES = 1, NO = 2)  LANGUAGE OF **ENGLISH** **LANGUAGE CODES:  QUESTIONNAIRE** 01 ENGLISH 03 LANGUAGE 3 05 LANGUAGE 5  02 LANGUAGE 2 04 LANGUAGE 4 06 LANGUAGE 6 | | | | | | |
| SUPERVISOR   \|  \|  \|  \|  \| \| --- \| --- \| --- \| --- \|   NAME NUMBER | | FIELD EDITOR   \|  \|  \|  \|  \| \| --- \| --- \| --- \| --- \|   NAME NUMBER | | | OFFICE EDITOR   \|  \|  \| \| --- \| --- \|   NUMBER | KEYED BY   \|  \|  \| \| --- \| --- \|   NUMBER |

(1) This section should be adapted for country-specific survey design.

Note: Brackets [ ] indicate items that should be adapted on a country-specific basis.

HH-1

THIS PAGE IS INTENTIONALLY BLANK

HH-2

HOUSEHOLD SCHEDULE

|  |  |  | | | **IF AGE 5 OR OLDER** | | | | | | | | | |  | | | | |
| --- | --- | --- | --- | --- | --- | --- | --- | --- | --- | --- | --- | --- | --- | --- | --- | --- | --- | --- | --- |
| LINE NO. |  |  | | | DISABILITY | | | | | | | | | |  | | | | |
|  | **26** | **27** | | | **28** | | | | |  | | **3029** | | | **31** | | | | |
|  | Does (NAME) wear glasses or contact lenses to help them see? | I would like to know if (NAME) has difficulty seeing even when wearing glasses or contact lenses. Would you say that (NAME) has no difficulty seeing, some difficulty, a lot of difficulty, or cannot see at all?   1. = NO DIFFICULTY SEEING 2. = SOME DIFFICULTY 3. = A LOT OF DIFFICULTY 4. = CANNOT SEE AT ALL   8 = DON'T KNOW | | | I would like to know if (NAME) has difficulty seeing. Would you say that (NAME) has no difficulty seeing, some difficulty, a lot of difficulty, or cannot see at all?   1. = NO DIFFICULTY SEEING 2. = SOME DIFFICULTY 3. = A LOT OF DIFFICULTY 4. = CANNOT SEE AT ALL   8 = DON'T KNOW | | | | | Does (NAME) wear a hearing aid? (1) |  | I would like to know if (NAME) has difficulty hearing even when using a hearing aid. Would you say that (NAME) has no difficulty hearing, some difficulty, a lot of difficulty, or cannot hear at all?  (1)   1. = NO DIFFICULTY HEARING 2. = SOME DIFFICULTY 3. = A LOT OF DIFFICULTY 4. = CANNOT HEAR AT ALL   8 = DON'T KNOW | | | I would like to know if (NAME) has difficulty hearing. Would you say that (NAME) has no difficulty hearing, some difficulty, a lot of difficulty, or cannot hear at all?   1. = NO DIFFICULTY HEARING 2. = SOME DIFFICULTY 3. = A LOT OF DIFFICULTY 4. = CANNOT HEAR AT ALL   8 = DON'T KNOW | | | | |
| 1 | Y N  1 2  GO TO 28 | 1 | 2 3 4  (GO TO 29) | 8 | 1 | 2 | 3 | 4 | 8 | Y  1  GO TO 31 | N  2 | 1 | 2 3 4  (GO TO 32) | 8 | 1 | 2 | 3 | 4 | 8 |
| 2 | 1 2  GO TO 28 | 1 | 2 3 4  (GO TO 29) | 8 | 1 | 2 | 3 | 4 | 8 | 1 2  GO TO 31 | | 1 | 2 3 4  (GO TO 32) | 8 | 1 | 2 | 3 | 4 | 8 |
| 3 | 1 2  GO TO 28 | 1 | 2 3 4  (GO TO 29) | 8 | 1 | 2 | 3 | 4 | 8 | 1 2  GO TO 31 | | 1 | 2 3 4  (GO TO 32) | 8 | 1 | 2 | 3 | 4 | 8 |
| 4 | 1 2  GO TO 28 | 1 | 2 3 4  (GO TO 29) | 8 | 1 | 2 | 3 | 4 | 8 | 1 2  GO TO 31 | | 1 | 2 3 4  (GO TO 32) | 8 | 1 | 2 | 3 | 4 | 8 |
| 5 | 1 2  GO TO 28 | 1 | 2 3 4  (GO TO 29) | 8 | 1 | 2 | 3 | 4 | 8 | 1 2  GO TO 31 | | 1 | 2 3 4  (GO TO 32) | 8 | 1 | 2 | 3 | 4 | 8 |
| 6 | 1 2  GO TO 28 | 1 | 2 3 4  (GO TO 29) | 8 | 1 | 2 | 3 | 4 | 8 | 1 2  GO TO 31 | | 1 | 2 3 4  (GO TO 32) | 8 | 1 | 2 | 3 | 4 | 8 |
| 7 | 1 2  GO TO 28 | 1 | 2 3 4  (GO TO 29) | 8 | 1 | 2 | 3 | 4 | 8 | 1 2  GO TO 31 | | 1 | 2 3 4  (GO TO 32) | 8 | 1 | 2 | 3 | 4 | 8 |
| 8 | 1 2  GO TO 28 | 1 | 2 3 4  (GO TO 29) | 8 | 1 | 2 | 3 | 4 | 8 | 1 2  GO TO 31 | | 1 | 2 3 4  (GO TO 32) | 8 | 1 | 2 | 3 | 4 | 8 |
| 9 | 1 2  GO TO 28 | 1 | 2 3 4  (GO TO 29) | 8 | 1 | 2 | 3 | 4 | 8 | 1 2  GO TO 31 | | 1 | 2 3 4  (GO TO 32) | 8 | 1 | 2 | 3 | 4 | 8 |
| 10 | 1 2  GO TO 28 | 1 | 2 3 4  (GO TO 29) | 8 | 1 2 3 4 8 | | | | | 1 2  GO TO 31 | | 1 | 2 3 4  (GO TO 32) | 8 | 1 2 3 4 8 | | | | |

HOUSEHOLD SCHEDULE

|  |  | | | | | **IF AGE 5 OR OLDER** | | | | | | | | | |  | | | | |
| --- | --- | --- | --- | --- | --- | --- | --- | --- | --- | --- | --- | --- | --- | --- | --- | --- | --- | --- | --- | --- |
| LINE NO. |  | | | | | DISABILITY | | | | | | | | | |  | | | | |
|  | **32** | | | | | **33** | | | | | **34** | | | | | **35** | | | | |
|  | I would like to know if (NAME) has difficulty communicating when using his/her usual language. Would you say that (NAME) has no difficulty understanding or being understood, some difficulty, a lot of difficulty, or cannot communicate at all? | | | | | I would like to know if (NAME) has difficulty remembering or concentrating. Would you say that (NAME) has no difficulty remembering or concentrating, some difficulty, a lot of difficulty, or cannot remember or concentrate at all? | | | | | I would like to know if (NAME) has difficulty walking or climbing steps. Would you say that (NAME) has no difficulty walking or climbing steps, some difficulty, a lot of difficulty, or cannot walk or climb steps at all? | | | | | I would like to know if (NAME) has difficulty washing all over or dressing. Would you say that (NAME) has no difficulty washing all over or dressing, some difficulty, a lot of difficulty, or cannot wash all over or dress at all? | | | | |
|  | 1 = NO DIFFICULTY | | | | | 1 = NO DIFFICULTY | | | | | 1 = NO DIFFICULTY WALKING | | | | | 1 = NO DIFFICULTY WASHING | | | | |
|  | COMMUNICATING | | | | | REMEMBERING/ | | | | | OR CLIMBING | | | | | OR DRESSING | | | | |
|  | 2 = SOME DIFFICULTY | | | | | CONCENTRATING | | | | | 2 = SOME DIFFICULTY | | | | | 2 = SOME DIFFICULTY | | | | |
|  | 3 = A LOT OF DIFFICULTY | | | | | 2 = SOME DIFFICULTY | | | | | 3 = A LOT OF DIFFICULTY | | | | | 3 = A LOT OF DIFFICULTY | | | | |
|  | 4 = CANNOT COMMUNICATE | | | | | 3 = A LOT OF DIFFICULTY | | | | | 4 = CANNOT WALK OR | | | |  | 4 = CANNOT WASH OR | | | |  |
|  | AT ALL | | |  |  | 4 = CANNOT REMEMBER/ | | | | | CLIMB AT ALL | | | |  | DRESS AT ALL | | | |  |
|  | 8 = DON'T KNOW | | |  |  | CONCENTRATE AT ALL 8 = DON'T KNOW | | |  |  | 8 = DON'T KNOW | | |  |  | 8 = DON'T KNOW | | |  |  |
| 1 | 1 | 2 | 3 | 4 | 8 | 1 | 2 | 3 | 4 | 8 | 1 | 2 | 3 | 4 | 8 | 1 | 2 | 3 | 4 | 8 |
| 2 | 1 | 2 | 3 | 4 | 8 | 1 | 2 | 3 | 4 | 8 | 1 | 2 | 3 | 4 | 8 | 1 | 2 | 3 | 4 | 8 |
| 3 | 1 | 2 | 3 | 4 | 8 | 1 | 2 | 3 | 4 | 8 | 1 | 2 | 3 | 4 | 8 | 1 | 2 | 3 | 4 | 8 |
| 4 | 1 | 2 | 3 | 4 | 8 | 1 | 2 | 3 | 4 | 8 | 1 | 2 | 3 | 4 | 8 | 1 | 2 | 3 | 4 | 8 |
| 5 | 1 | 2 | 3 | 4 | 8 | 1 | 2 | 3 | 4 | 8 | 1 | 2 | 3 | 4 | 8 | 1 | 2 | 3 | 4 | 8 |
| 6 | 1 | 2 | 3 | 4 | 8 | 1 | 2 | 3 | 4 | 8 | 1 | 2 | 3 | 4 | 8 | 1 | 2 | 3 | 4 | 8 |
| 7 | 1 | 2 | 3 | 4 | 8 | 1 | 2 | 3 | 4 | 8 | 1 | 2 | 3 | 4 | 8 | 1 | 2 | 3 | 4 | 8 |
| 8 | 1 | 2 | 3 | 4 | 8 | 1 | 2 | 3 | 4 | 8 | 1 | 2 | 3 | 4 | 8 | 1 | 2 | 3 | 4 | 8 |
| 9 | 1 | 2 | 3 | 4 | 8 | 1 | 2 | 3 | 4 | 8 | 1 | 2 | 3 | 4 | 8 | 1 | 2 | 3 | 4 | 8 |
| 10 | 1 2 3 4 8 | | | | | 1 2 3 4 8 | | | | | 1 2 3 4 8 | | | | | 1 2 3 4 8 | | | | |

HOUSEHOLD SCHEDULE

|  |  |  | | | **IF AGE 5 OR OLDER** | | | | | | | | |  | | | | |
| --- | --- | --- | --- | --- | --- | --- | --- | --- | --- | --- | --- | --- | --- | --- | --- | --- | --- | --- |
| LINE NO. |  |  | | | DISABILITY | | | | | | | | |  | | | | |
|  | **26** | **27** | | | **28** | | | | |  | **3029** | | | **31** | | | | |
|  | Does (NAME) wear glasses or contact lenses to help them see? | I would like to know if (NAME) has difficulty seeing even when wearing glasses or contact lenses. Would you say that (NAME) has no difficulty seeing, some difficulty, a lot of difficulty, or cannot see at all?   1. = NO DIFFICULTY SEEING 2. = SOME DIFFICULTY 3. = A LOT OF DIFFICULTY 4. = CANNOT SEE AT ALL   8 = DON'T KNOW | | | I would like to know if (NAME) has difficulty seeing. Would you say that (NAME) has no difficulty seeing, some difficulty, a lot of difficulty, or cannot see at all?   1. = NO DIFFICULTY SEEING 2. = SOME DIFFICULTY 3. = A LOT OF DIFFICULTY 4. = CANNOT SEE AT ALL   8 = DON'T KNOW | | | | | Does (NAME) wear a hearing aid? (1) | I would like to know if (NAME) has difficulty hearing even when using a hearing aid. Would you say that (NAME) has no difficulty hearing, some difficulty, a lot of difficulty, or cannot hear at all?  (1)   1. = NO DIFFICULTY HEARING 2. = SOME DIFFICULTY 3. = A LOT OF DIFFICULTY 4. = CANNOT HEAR AT ALL   8 = DON'T KNOW | | | I would like to know if (NAME) has difficulty hearing. Would you say that (NAME) has no difficulty hearing, some difficulty, a lot of difficulty, or cannot hear at all?   1. = NO DIFFICULTY HEARING 2. = SOME DIFFICULTY 3. = A LOT OF DIFFICULTY 4. = CANNOT HEAR AT ALL   8 = DON'T KNOW | | | | |
|  | Y N |  | |  |  | | |  |  |  |  | |  |  | | |  |  |
| 11 | 1 2 | 1 | 2 3 4 | 8 | 1 | 2 | 3 | 4 | 8 | 1 2 | 1 | 2 3 4 | 8 | 1 | 2 | 3 | 4 | 8 |
|  | GO TO 28 |  | (GO TO 29) |  |  |  |  |  |  | GO TO 31 |  | (GO TO 32) |  |  |  |  |  |  |
| 12 | 1 2  GO TO 28 | 1 | 2 3 4  (GO TO 29) | 8 | 1 | 2 | 3 | 4 | 8 | 1 2  GO TO 31 | 1 | 2 3 4  (GO TO 32) | 8 | 1 | 2 | 3 | 4 | 8 |
| 13 | 1 2  GO TO 28 | 1 | 2 3 4  (GO TO 29) | 8 | 1 | 2 | 3 | 4 | 8 | 1 2  GO TO 31 | 1 | 2 3 4  (GO TO 32) | 8 | 1 | 2 | 3 | 4 | 8 |
| 14 | 1 2  GO TO 28 | 1 | 2 3 4  (GO TO 29) | 8 | 1 | 2 | 3 | 4 | 8 | 1 2  GO TO 31 | 1 | 2 3 4  (GO TO 32) | 8 | 1 | 2 | 3 | 4 | 8 |
| 15 | 1 2  GO TO 28 | 1 | 2 3 4  (GO TO 29) | 8 | 1 | 2 | 3 | 4 | 8 | 1 2  GO TO 31 | 1 | 2 3 4  (GO TO 32) | 8 | 1 | 2 | 3 | 4 | 8 |
| 16 | 1 2  GO TO 28 | 1 | 2 3 4  (GO TO 29) | 8 | 1 | 2 | 3 | 4 | 8 | 1 2  GO TO 31 | 1 | 2 3 4  (GO TO 32) | 8 | 1 | 2 | 3 | 4 | 8 |
| 17 | 1 2  GO TO 28 | 1 | 2 3 4  (GO TO 29) | 8 | 1 | 2 | 3 | 4 | 8 | 1 2  GO TO 31 | 1 | 2 3 4  (GO TO 32) | 8 | 1 | 2 | 3 | 4 | 8 |
| 18 | 1 2  GO TO 28 | 1 | 2 3 4  (GO TO 29) | 8 | 1 | 2 | 3 | 4 | 8 | 1 2  GO TO 31 | 1 | 2 3 4  (GO TO 32) | 8 | 1 | 2 | 3 | 4 | 8 |
| 19 | 1 2  GO TO 28 | 1 | 2 3 4  (GO TO 29) | 8 | 1 | 2 | 3 | 4 | 8 | 1 2  GO TO 31 | 1 | 2 3 4  (GO TO 32) | 8 | 1 | 2 | 3 | 4 | 8 |
| 20 | 1 2  GO TO 28 | 1 | 2 3 4  (GO TO 29) | 8 | 1 2 3 4 8 | | | | | 1 2  GO TO 31 | 1 | 2 3 4  (GO TO 32) | 8 | 1 2 3 4 8 | | | | |

HOUSEHOLD SCHEDULE

|  |  | | | | | **IF AGE 5 OR OLDER** | | | | | | | | | |  | | | | |
| --- | --- | --- | --- | --- | --- | --- | --- | --- | --- | --- | --- | --- | --- | --- | --- | --- | --- | --- | --- | --- |
| LINE NO. |  | | | | | DISABILITY | | | | | | | | | |  | | | | |
|  | **32** | | | | | **33** | | | | | **34** | | | | | **35** | | | | |
|  | I would like to know if (NAME) has difficulty communicating when using his/her usual language. Would you say that (NAME) has no difficulty understanding or being understood, some difficulty, a lot of difficulty, or cannot communicate at all?   1. = NO DIFFICULTYCOMMUNICATING 2. = SOME DIFFICULTY 3. = A LOT OF DIFFICULTY 4. = CANNOT COMMUNICATEAT ALL   8 = DON'T KNOW | | | | | I would like to know if (NAME) has difficulty remembering or concentrating. Would you say that (NAME) has no difficulty remembering or concentrating, some difficulty, a lot of difficulty, or cannot remember or concentrate at all?   1. = NO DIFFICULTY REMEMBERING/   CONCENTRATING   1. = SOME DIFFICULTY 2. = A LOT OF DIFFICULTY 3. = CANNOT REMEMBER/ CONCENTRATE AT ALL 8 = DON'T KNOW | | | | | I would like to know if (NAME) has difficulty walking or climbing steps. Would you say that (NAME) has no difficulty walking or climbing steps, some difficulty, a lot of difficulty, or cannot walk or climb steps at all?   1. = NO DIFFICULTY WALKING   OR CLIMBING   1. = SOME DIFFICULTY 2. = A LOT OF DIFFICULTY 3. = CANNOT WALK OR   CLIMB AT ALL  8 = DON'T KNOW | | | | | I would like to know if (NAME) has difficulty washing all over or dressing. Would you say that (NAME) has no difficulty washing all over or dressing, some difficulty, a lot of difficulty, or cannot wash all over or dress at all?   1. = NO DIFFICULTY WASHING   OR DRESSING   1. = SOME DIFFICULTY 2. = A LOT OF DIFFICULTY4 = CANNOT WASH OR DRESS AT ALL 8 = DON'T KNOW | | | | |
| 11 | 1 | 2 | 3 | 4 | 8 | 1 | 2 | 3 | 4 | 8 | 1 | 2 | 3 | 4 | 8 | 1 | 2 | 3 | 4 | 8 |
| 12 | 1 | 2 | 3 | 4 | 8 | 1 | 2 | 3 | 4 | 8 | 1 | 2 | 3 | 4 | 8 | 1 | 2 | 3 | 4 | 8 |
| 13 | 1 | 2 | 3 | 4 | 8 | 1 | 2 | 3 | 4 | 8 | 1 | 2 | 3 | 4 | 8 | 1 | 2 | 3 | 4 | 8 |
| 14 | 1 | 2 | 3 | 4 | 8 | 1 | 2 | 3 | 4 | 8 | 1 | 2 | 3 | 4 | 8 | 1 | 2 | 3 | 4 | 8 |
| 15 | 1 | 2 | 3 | 4 | 8 | 1 | 2 | 3 | 4 | 8 | 1 | 2 | 3 | 4 | 8 | 1 | 2 | 3 | 4 | 8 |
| 16 | 1 | 2 | 3 | 4 | 8 | 1 | 2 | 3 | 4 | 8 | 1 | 2 | 3 | 4 | 8 | 1 | 2 | 3 | 4 | 8 |
| 17 | 1 | 2 | 3 | 4 | 8 | 1 | 2 | 3 | 4 | 8 | 1 | 2 | 3 | 4 | 8 | 1 | 2 | 3 | 4 | 8 |
| 18 | 1 | 2 | 3 | 4 | 8 | 1 | 2 | 3 | 4 | 8 | 1 | 2 | 3 | 4 | 8 | 1 | 2 | 3 | 4 | 8 |
| 19 | 1 | 2 | 3 | 4 | 8 | 1 | 2 | 3 | 4 | 8 | 1 | 2 | 3 | 4 | 8 | 1 | 2 | 3 | 4 | 8 |
| 20 | 1 2 3 4 8 | | | | | 1 2 3 4 8 | | | | | 1 2 3 4 8 | | | | | 1 2 3 4 8 | | | | |

SECTION DB. FOOTNOTES

(1) This question may be excluded in countries where wearing a hearing aid is not common.

HH-7

# Disability Module Interviewer Instructions

**DISABILITIES (Qs. 26 – 35)**

## Columns 26 through 35: DISABILITIES

Disability is an umbrella term for impairments, activity limitations and participation restrictions. Disability is impossible to describe in one or two sentences, partly because it covers a huge range of things and also touches a large number of people. It is likely to affect everyone at some stage in their lives. Generally, a disability is inability or great difficulty in performing one or more major life activities in the person’s current social environment, either because of a physical, mental, or psychological illness, or an impairment with any part of the body, such as a missing, or damaged part of the body.

Major life activities include the following:

- Having a full range of movement while standing, lifting, walking and so forth,
- Having intact senses (vision, hearing, touch, smell, taste, balance),
- Communicating with others (speaking and writing),
- Learning and working,
- Caring for oneself in hygiene and homemaking,
- Using mental processes such as thinking, concentrating, and problem solving,
- Interacting with others and developing and maintaining relationships.

Questions 26 through 35 are asked for each household member and visitor who spent the night before the survey (column 5 and/or column 6 = YES) and who are age 5 and older. The questions ask about *difficulty* with vision, hearing, communicating/understanding, memory or concentration, walking, and the capacity to undertake basic activities such as washing or dressing. If a person is experiencing difficulty in any of the areas, information is sought about the degree of difficulty that he/she has.

Having *difficulty* with an activity means:

- Being unable to perform the function
- Increased effort to perform the function
- Discomfort or pain when performing the function
- Slowness in performing the function

The answer categories are set up so that the respondent can indicate the level of difficulty.

1. NO DIFFICULTY
2. SOME DIFFICULTY
3. A LOT OF DIFFICULTY
4. CANNOT DO AT ALL

8. DON’T KNOW

Each question starts with an introduction asking about specific functions.

## Columns 26 - 28: SEEING

People with difficulty seeing may be born with this difficulty or acquire one later in life. It is important to note that most people who are registered blind may retain partial sight; only a very small percentage are totally blind. People who have difficulty seeing may have different degrees of sight and their difficulty may differ. Some people may have blurred vision, or may not be able to judge distances and speed, or to distinguish between objects that have a similar color or shape. Others who have difficulty seeing may only see things that are very close, or have a restricted range of vision, such as tunnel vision and no peripheral vision.

The purpose of this question is to identify persons who have vision difficulties or problems of any kind even when wearing glasses or contact lenses (if they wear glasses/contacts). They can have a problem seeing things close up or far away. They may not be able to see out of one eye or they may be only able to see directly in front of them, but not to the sides. Any difficulty with vision that they consider a problem should be captured.

**Seeing** refers to an individual’s capacity to perceive or observe what is happening around them.

If a respondent is hesitant about the degree of difficulty a household member/visitor experiences, ask them for their best judgment.

## Columns 29 - 31: HEARING

The purpose of these questions is to identify persons who have some hearing limitation or problems of any kind with their hearing even when using a hearing aid (if they wear a hearing aid). They can have a problem hearing only when they are in a noisy environment, or they may have problems distinguishing sounds from different sources. They may not be able to hear in one ear or both. Any difficulty with hearing that they consider a problem should be captured.

**Hearing** refers to an individual’s capacity to know what is being said to them or the sounds of activity, including danger, that is happening around them.

## Column 32: COMMUNICATING: UNDERSTANDING AND BEING UNDERSTOOD

Some people with speech and language disabilities may have difficulties in articulating sounds or understanding and formulating thoughts in spoken words. These difficulties may be immediately perceptible, or they may not emerge until discussion focuses on more abstract matters. Difficulties in speech and language may be associated with particular conditions, e.g. when the facial muscles are affected, as in stroke and cerebral palsy.

The purpose of this item is to identify persons who have some problems with talking, listening or understanding speech. They can have a problem making themselves understood, or the problem may be that they can’t understand people who talk to them or try to communicate with them in other ways. Sometimes they can be understood by members of the household who are familiar with them, but have difficulty to be understood outside the household.

**Communicating** refers to a person exchanging information or ideas with other people through the use of language. They may use their voices for their exchange or make signs or write the information they want to exchange. Communication can be interrupted at numerous places in the exchange process. It may involve mechanical problems such as hearing impairment or speech impairment, or it may be related to the ability of the mind to interpret the sounds that the auditory system is gathering and to recognize the words that are being used.

## Column 33: REMEMBERING AND CONCENTRATING

The purpose of this question is to identify household members or visitors age 5 and older who have some problems with remembering or concentrating. They can have a problem finding their way around, or the problem can be that they can’t concentrate on what they are doing, or they may forget where they are or what month it is. They may not remember what someone just said to them or they may seem confused or frightened about most things. Any difficulty with remembering, concentrating or understanding what is going on around them should be captured. We do not intend to capture difficulties remembering or concentrating because of common everyday situations such as high workload or stress, or as a result of substance abuse.

**Remembering** refers to an individual using his/her memory capacity in order to recall what has happened around them. It means the individual can bring to mind or think again about something that has taken place in the past (either the recent past or further back). In connection with younger people, remembering is often associated with storing facts learned in school and being able to retrieve them when needed.

**Concentrating** refers to an individual using his mental ability to accomplish some task such as reading, calculating numbers, learning something. It is associated with focusing on the task at hand in order to complete the task. It is the act of directing ones full attention to one subject or to focus without distraction on one thing.

## Column 34: WALKING, CLIMBING STEPS

The causes of difficulties walking or climbing steps might have started at birth or might be acquired later in life. The causes can vary widely, and can affect the person's bones, muscles, joints, nerves, tendons, spinal cord, sensory organs, and/or the brain. The condition leading to the difficulty can be stable or degenerative (as in the case of multiple sclerosis) and may render individuals unable to perform the same range of physical activities that those without these difficulties do and, consequently, they may face barriers of access.

The purpose of this question is to identify persons who have some limitation or problems of any kind getting around on foot. It may or may not contribute to difficulty in doing their daily activities. They can have a problem walking more than a block, or short or long distances, or the problem can be that they can’t walk up or down steps without difficulty. They may not be able to walk any distance without stopping to rest or they may not be able to walk without using some type of device such as a cane, a walker, or crutches. In some instances, they may be totally unable to stand for more than a minute or two and need a wheelchair to get from place to place.

In addition to problems with the musculoskeletal system, difficulties walking can include those resulting from impairments in balance, vision, endurance, or other systems. Any difficulty with walking (whether it is on flat land or up or down steps) should be captured.

**Walking** refers to an individual using his/her legs in such a way as to propel themselves over the ground to get from point A to point B. The capacity to walk should be without assistance of any device or human. If such assistance is needed, the person has difficulty walking.

## Column 35: WASHING and DRESSING

The purpose of this question is to identify persons who have difficulty with taking care of themselves independently. Washing and dressing represent tasks that occur on a daily basis and are basic activities. Note if the person is using an assistive device or has a person to help them with this function, it is highly likely they have difficulty with self-care.

**Washing all over** refers to the process of cleaning one’s entire body (usually with soap and water) in the usual manner for the culture. The washing activity includes cleaning hair and feet, as well as gathering any age and culturally appropriate items necessary for bathing such as soap or shampoo, a wash cloth, or water.

**Dressing** refers to all aspects of putting clothing or garments on the upper and lower body including the feet if culturally appropriate. Gathering clothing from storage areas (i.e. closet, dressers), securing buttons, tying knots, opening and closing zippers, etc., are example of aspects of dressing that could be considered by the respondent. The aspects of dressing should be age and culturally appropriate.

End of DB instructions.

Table DB.1 Disability by domain and age

Table DB.2.1 Disability among adults according to background characteristics: Women

Table DB.2.2 Disability among adults according to background characteristics: Men

**Table DB.1 Disability by domain and age**

Percent distribution of de facto household population age 5 and over by the degree of difficulty in functioning according to domain, and percent distribution by the highest degree of difficulty in functioning in at least one domain by age, [Country Survey Year]

A lot of difficulty or

Degree of difficulty

| Domain and age | No difficulty | Some  difficulty | A lot of difficulty | Cannot do at all | Don't know/ missing | Total | cannot do at all | Number of persons |
| --- | --- | --- | --- | --- | --- | --- | --- | --- |
| **Domain**  Difficulty seeing |  |  |  |  |  | 100.0 |  |  |
| Difficulty hearing |  |  |  |  |  | 100.0 |  |  |
| Difficulty communicating  Difficulty remembering or concentrating |  |  |  |  |  | 100.0 |  |  |
| Difficulty walking or climbing steps |  |  |  |  |  | 100.0 |  |  |
| Difficulty washing all over or dressing |  |  |  |  |  | 100.0 |  |  |
| **Difficulty in at least one domain^1^**  5-9 |  |  |  |  |  | 100.0 |  |  |
| 10-14 |  |  |  |  |  | 100.0 |  |  |
| 15-19 |  |  |  |  |  | 100.0 |  |  |
| 20-29 |  |  |  |  |  | 100.0 |  |  |
| 30-39 |  |  |  |  |  | 100.0 |  |  |
| 40-49 |  |  |  |  |  | 100.0 |  |  |
| 50-59 |  |  |  |  |  | 100.0 |  |  |
| 60+ |  |  |  |  |  | 100.0 |  |  |
| Age 15 and over |  |  |  |  |  | 100.0 |  |  |
| Total |  |  |  |  |  | 100.0 |  |  |
| 1 If a person was reported to have difficulty in more than one domain, only the highest level of difficulty is shown. | | | | | |  |  |  |

### Table DB.2.1 Disability among adults according to background characteristics: Women

Percentage of the de facto household population age 15 and over who have difficulty in functioning according to domain, by the highest degree of difficulty in at least one domain, and percentage with a lot of difficulty or cannot do at all in more than one domain, according to background characteristics, [Country Survey Year]

Some difficulty, a lot of difficulty, or cannot do at all Difficulty in at least one domain^1^ A lot of

Rememberin difficulty or g A lot of cannot do at

No difficulty or Walking or Washing all difficulty or all in more

in any Communic- concentratin climbing over or Don't know/ Some A lot of Cannot do at cannot do at than one Number of

| Background characteristic | domain | Seeing | Hearing | ating g | steps dressing | missing | difficulty | difficulty | all | all | domain | persons |
| --- | --- | --- | --- | --- | --- | --- | --- | --- | --- | --- | --- | --- |
|  |  |  |  |  | WOMEN |  |  |  |  |  |  |  |
| **Marital status**  Never married  Married  Widowed  Divorced  **Residence** Urban  Rural  **Region**  Region 1  Region 2  Region 3  Region 4  **Education**  No education  Primary  Secondary  More than secondary  **Wealth quintile**  Lowest  Second  Middle  Fourth  Highest    Total |  |  |  |  |  |  |  |  |  |  |  |  |
| ^1^ If a person was reported to have difficulty in more than one domain, only the highest level of difficulty is shown. | | | | | |  |  |  |  |  |  |  |

### Table DB.2.2 Disability among adults according to background characteristics: Men

Percentage of the de facto household population age 15 and over who have difficulty in functioning according to domain, by the highest degree of difficulty in at least one domain, and percentage with a lot of difficulty or cannot do at all in more than one domain, according to background characteristics, [Country Survey Year]

Some difficulty, a lot of difficulty, or cannot do at all Difficulty in at least one domain^1^ A lot of

Rememberin difficulty or g A lot of cannot do at

No difficulty or Walking or Washing all difficulty or all in more

in any Communic- concentratin climbing over or Don't know/ Some A lot of Cannot do at cannot do at than one Number of

| Background characteristic | domain | Seeing | Hearing | ating g | steps | dressing | missing | difficulty | difficulty | all | all | domain | persons |
| --- | --- | --- | --- | --- | --- | --- | --- | --- | --- | --- | --- | --- | --- |
|  |  |  |  |  |  | MEN |  |  |  |  |  |  |  |
| **Marital status**  Never married  Married  Widowed  Divorced  **Residence** Urban  Rural  **Region**  Region 1  Region 2  Region 3  Region 4  **Education**  No education  Primary  Secondary  More than secondary  **Wealth quintile**  Lowest  Second  Middle  Fourth  Highest    Total |  |  |  |  |  |  |  |  |  |  |  |  |  |
| ^1^ If a person was reported to have difficulty in more than one domain, only the highest level of difficulty is shown. | | | | | |  |  |  |  |  |  |  |  |
